# Rapid Assessment of Genetic Ancestry in Populations of Unknown Origin by Genome-Wide Genotyping of Pooled Samples

**DOI:** 10.1371/journal.pgen.1000866

**Published:** 2010-03-05

**Authors:** Charleston W. K. Chiang, Zofia K. Z. Gajdos, Joshua M. Korn, Finny G. Kuruvilla, Johannah L. Butler, Rachel Hackett, Candace Guiducci, Thutrang T. Nguyen, Rainford Wilks, Terrence Forrester, Christopher A. Haiman, Katherine D. Henderson, Loic Le Marchand, Brian E. Henderson, Mark R. Palmert, Colin A. McKenzie, Helen N. Lyon, Richard S. Cooper, Xiaofeng Zhu, Joel N. Hirschhorn

**Affiliations:** 1 Department of Genetics, Harvard Medical School, Boston, Massachusetts, United States of America; 2 Program in Medical and Population Genetics, Broad Institute of Harvard and Massachusetts Institute of Technology, Cambridge, Massachusetts, United States of America; 3 Program in Genomics and Divisions of Genetics and Endocrinology, Children's Hospital, Boston, Massachusetts, United States of America; 4 Center for Human Genetic Research, Massachusetts General Hospital, Boston, Massachusetts, United States of America; 5 Department of Molecular Biology, Massachusetts General Hospital, Boston, Massachusetts, United States of America; 6 Epidemiology Research Unit, Tropical Medicine Research Institute, University of the West Indies, Kingston, Jamaica; 7 Tropical Metabolism Research Unit, Tropical Medicine Research Institute, University of the West Indies, Kingston, Jamaica; 8 Department of Preventive Medicine, Keck School of Medicine, University of Southern California, Los Angeles, California, United States of America; 9 Department of Population Sciences, Division of Cancer Etiology, City of Hope National Medical Center, Duarte, California, United States of America; 10 Epidemiology Program, Cancer Research Center of Hawaii, University of Hawaii, Honolulu, Hawaii, United States of America; 11 Division of Endocrinology, The Hospital for Sick Children, Toronto, Ontario, Canada; 12 Department of Pediatrics, University of Toronto, Toronto, Ontario, Canada; 13 Department of Preventive Medicine and Epidemiology, Stritch School of Medicine, Loyola University Chicago, Maywood, Illinois, United States of America; 14 Department of Biostatistics and Epidemiology, Case Western Reserve University, Cleveland, Ohio, United States of America; University of Arizona, United States of America

## Abstract

As we move forward from the current generation of genome-wide association (GWA) studies, additional cohorts of different ancestries will be studied to increase power, fine map association signals, and generalize association results to additional populations. Knowledge of genetic ancestry as well as population substructure will become increasingly important for GWA studies in populations of unknown ancestry. Here we propose genotyping pooled DNA samples using genome-wide SNP arrays as a viable option to efficiently and inexpensively estimate admixture proportion and identify ancestry informative markers (AIMs) in populations of unknown origin. We constructed DNA pools from African American, Native Hawaiian, Latina, and Jamaican samples and genotyped them using the Affymetrix 6.0 array. Aided by individual genotype data from the African American cohort, we established quality control filters to remove poorly performing SNPs and estimated allele frequencies for the remaining SNPs in each panel. We then applied a regression-based method to estimate the proportion of admixture in each cohort using the allele frequencies estimated from pooling and populations from the International HapMap Consortium as reference panels, and identified AIMs unique to each population. In this study, we demonstrated that genotyping pooled DNA samples yields estimates of admixture proportion that are both consistent with our knowledge of population history and similar to those obtained by genotyping known AIMs. Furthermore, through validation by individual genotyping, we demonstrated that pooling is quite effective for identifying SNPs with large allele frequency differences (i.e., AIMs) and that these AIMs are able to differentiate two closely related populations (HapMap JPT and CHB).

## Author Summary

Many association studies have been published looking for genetic variants contributing to a variety of human traits such as obesity, diabetes, and height. Because the frequency of genetic variants can differ across populations, it is important to have estimates of genetic ancestry in the individuals being studied. In this study, we were able to measure genetic ancestry in populations of mixed ancestry by genotyping pooled, rather than individual, DNA samples. This represents a rapid and inexpensive means for modeling genetic ancestry and thus could facilitate future association or population-genetic studies in populations of unknown ancestry for which whole-genome data do not already exist.

## Introduction

Genetic ancestry, as studied through DNA sequence variation, has shed light on the history, migration patterns, and relationships among human populations [1],[2]. In the context of medical population genetics, genetic ancestry forms the basis of admixture mapping [3]. Additionally, genetic ancestry is useful for proper matching of cases and controls and is also an important covariate to consider in association studies for complex human traits [4],[5] as spurious associations around variants with large allele frequency differences between populations have long been recognized as potential confounders [6]–[9]. For admixed populations, having an estimated proportion of genetic ancestry attributable to each ancestral population (*i.e.*, the admixture proportion) would also allow the construction of weighted reference panels, which has been shown to enable a more efficient design of a panel of tag SNPs to capture untyped variations over a genomic region (*e.g.*, a candidate gene region) and possibly facilitate more efficient imputation of untyped SNPs genome-wide in admixed populations [10]. Moreover, as we move forward from hypothesis-generating genome-wide association (GWA) studies, the research focus will start to shift to fine mapping of associated signals and/or pathways identified through such studies and will also expand to include understudied diseases as well as studies in additional populations of unknown ancestry. For all of these studies, knowledge of genetic ancestry (and thus potential population substructure) will be necessary.

Currently, two main approaches exist for inferring genetic ancestry. If the ancestral populations of the population being studied are known, ancestry informative markers (AIMs) numbering in the hundreds can be genotyped to infer global ancestry via principal components analysis (PCA) or a clustering-based algorithm (for examples, see [8]–[16]). However, often the ancestral populations are not known with confidence, and many markers would need to be genotyped in the discovery phase to assemble a panel of AIMs. Moreover, AIMs identified in this manner will only be informative for the axis of ancestry they are selected to explain (*e.g.*, a panel of AIMs selected to differentiate between Africans and Europeans will be less effective for differentiating northern Europeans from southern Europeans). The alternative approach is to apply PCA to individual-level genetic data for a large number of loci, typically obtained from GWA studies, to infer global ancestry. The limitation of this approach is the high cost of obtaining genome-wide genotype data from a sizable cohort, particularly when studying a less well-funded phenotype. Therefore, the need to efficiently (both in terms of cost and time) assess the biogeographical ancestry in the study population and to rapidly screen hundreds of thousands of genetic makers for AIMs will be valuable for future genetic association and demographic studies. This is particularly true for populations of relatively complicated admixture or of origins dissimilar to standard reference populations such as those catalogued by the International HapMap Consortium [17]. One possible method for rapidly and inexpensively estimating admixture proportion and identifying AIMs in a cohort is through genotyping of pooled DNA.

Genotyping pools of DNA from multiple individuals rather than genotyping each individual separately has been proposed as a cost-effective alternative to GWA studies (see [18]). One study estimated that a 20-fold reduction in cost could theoretically be achieved if pooled genotyping were employed [19]. This reduction in cost would allow preliminary GWA studies of numerous orphan diseases to be conducted. For this reason, several reports have investigated the feasibility of and have developed analysis tools for genotyping pooled DNA using SNP microarrays (see [19]–[26], among others). Despite the potential cost-savings of pooled genotyping, drawbacks of not directly measuring individual genotypes include loss of the ability to study additional or sub- phenotypes within the pooled cohort and loss of the ability to detect gene-gene interactions (see [20]). It has also not been shown definitively that small allele frequency differences between cases and controls can be reliably detected given the additional imprecision in allele frequency estimates due to pooling. Indeed, reproducible associations have only been reported for variants with large effect sizes (for example, [20], [27]–[30]), whereas common variants known to be associated with common diseases such as type 2 diabetes and obesity typically have modest effect sizes with odds ratios ranging from about 1.1 to 1.3 [31].

Because pooled genotyping may reliably detect SNPs with large between-group allele frequency differences [20], [27]–[30], we hypothesized that this approach may represent a feasible method to identify AIMs, as these are, by definition, markers that display large allele frequency differences between two populations. To test this hypothesis, we constructed four pools from African American samples and genotyped both the pooled and individual DNA samples at ∼900 K markers using the Affymetrix 6.0 array. Taking advantage of the expected allele frequency estimates based on individual genotypes, we established a set of quality control (QC) filters to enrich for SNPs truly displaying allele frequency differences between two pools and applied QC filter to a Hawaiian cohort, a Latina cohort, and two Jamaican cohorts that had been similarly pooled and genotyped. Then, based on the estimated allele frequencies for post-QC SNPs, we were able to reliably estimate admixture proportions in these pooled cohorts from admixed populations, using HapMap reference panels as proxies for the populations ancestral to the admixed populations. Moreover, we were able to identify AIMs informative for ancestry beyond what can be modeled by the HapMap reference panels. Therefore, genome-wide genotyping of pooled DNA appears to be extremely efficient and informative for assessing the genetic ancestry of a population.

## Results

### DNA pool construction and quality control filters

**Figure 1:**
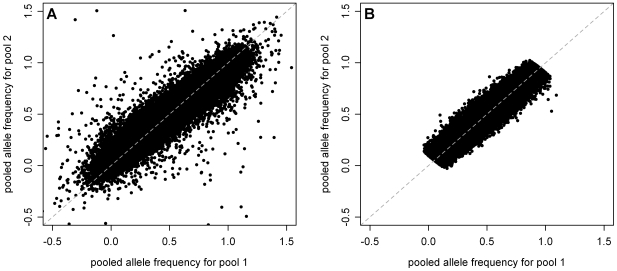
Estimated allele frequencies in MEC-H pool 1 versus MEC-H pool 2 before and after application of QC filters. Estimated allele frequencies for 100,000 random SNPs from the two MEC-H pools were plotted against each other (A) before SNP QC filtering and (B) after applying all four SNP QC filters. There were ∼869 K autosomal SNPs pre-QC filtering, and ∼306 K SNPs post-QC filtering (see Methods). Among the 5,000 SNPs with the largest AF differences between the two pools, the mean AF difference in the post-QC filtered dataset was significantly reduced (0.604 pre-QC versus 0.186 post-QC, *P*≪10^−15^ by unpaired two-tailed t-test). Note that this comparison is based only on the average of allele frequency estimates, without taking into account the error involved in such estimates, which is compensated for when calculating the association χ^2^ statistic (see Methods).

In total we constructed four DNA pools of 521 African American samples from Maywood, IL (MAY); two pools of 321 African American women (MEC-AA), two pools of 252 Native Hawaiian women (MEC-H), two pools of 332 Latina women (MEC-L), and two pools of 202 Japanese American women (MEC-J) from Los Angeles, CA and Honolulu, HI; six pools of 688 Jamaican samples from Kingston, Jamaica (GXE); and four pools of 480 Jamaican samples from Spanishtown, Jamaica (SPT) (see Text S1 for details). Each pool was genotyped in triplicate using the Affymetrix 6.0 array. Samples comprising the MAY panel were also genotyped individually as part of a separate GWA study of obesity (C.W.K.C., H.N.L., R.S.C., X.Z., and J.N.H., unpublished). For each pool, pooled allele frequencies (AF) were estimated as the proportion of angular distance observed for the pooled sample relative to that observed for the individual samples on the same plate, and averaged over all replicates (see Methods for details). Quality control (QC) was performed in two stages. First, any pool replicate with excessively low intensity, low call rate, or high heterozygosity compared to the other replicates within the same pool was either re-genotyped or dropped from the study (see Text S1). Second, because of the availability of individual genotype data for the MAY panel, it was used as a training set to establish a set of four SNP QC filters to preferentially eliminate SNPs that genotyped poorly or inconsistently (see Methods, Text S1). ∼306 K SNPs in MEC-H, ∼359 K SNPs in MEC-L, ∼346 K SNPs in MEC-J, ∼477 K SNPs in MEC-AA, ∼353 K SNPs in GXE, and ∼307 K SNPs in SPT passed all four QC filters. When examining the correlation of the estimated allele frequencies of one of the MEC-H pools with those of the other MEC-H pool the SNP QC filters were effective in removing the vast majority of SNPs predicted to have large allele frequency differences, even though the difference in predicted AF between the two pools was not part of the QC filter (Figure 1A and 1B). Similar results were observed for the pools from other panels (data not shown). These removed SNPs are likely to be false positives, as very few SNPs with large AF differences between two samplings from the same underlying population are expected. The effectiveness of the filters in removing poorly genotyped SNPs is also evident when comparing the estimated allele frequency by pooling to the actual allele frequency by individual genotyping in the MAY panel (Figure S1). We attempted to identify putative AIMs only among the SNPs that passed the QC filters (below).

### Estimation of admixture proportion

To both assess genetic ancestry and identify new AIMs particular to the admixed populations, we first used our QC-filtered pooled genotype data to estimate the relative contributions of different continental ancestries to each of our admixed populations (MEC-H, MEC-L, MEC-AA, GXE, and SPT). Second, for each of our admixed panels we constructed a corresponding weighted reference panel (pseudopopulation) based on the estimated admixture proportion, and identified putative AIMs, *i.e.,* SNPs with pooled AF estimates significantly different from those predicted by the pseudopopulation. Finally, we validated putative AIMs by genotyping the individuals who comprised the pools.

To estimate the proportion of ancestry relative to the HapMap reference panels (*i.e.*, the admixture proportion), we applied a linear regression-based approach to the QC-filtered data, overcoming the uncertainty in pooled AF estimates with the high density of SNPs. For each SNP, we modeled the estimated allele frequency of the pooled sample as a linear combination of the known allele frequencies in the HapMap YRI (West African), CEU (European), and/or CHB/JPT (East Asian) reference panels. The associated regression coefficients can be thought of as estimates of the proportional contribution from each of the reference panels (see Methods). We first tested the method in a population of known ancestry. For the MAY pool, regression estimates from pooling yielded an estimated overall admixture proportion of ∼82.4% YRI and ∼17.5% CEU (Table 1). This estimate is very similar to that obtained using allele frequencies based on individual genotyping on pre- or post- QC-filtered SNPs (∼81.2% YRI and ∼17.8% CEU pre-QC, ∼80.6% YRI and ∼17.6% CEU post-QC), showing that the method is robust to pooling-associated error in estimating allele frequencies. Additionally, this estimate is also very close to that obtained when we restricted the analysis to genotypes at 699 published ancestry informative markers (AIMs) found on the Affymetrix 6.0 array [32] and estimated ancestry using STRUCTURE [13] (∼83.3% YRI and ∼16.7% CEU, Table 1), and previously published estimates (∼81.2% YRI and ∼18.8% CEU [33]; ∼80.5% YRI and ∼19.5% CEU [34]) for this population.

**Table 1:** Comparison of estimates of admixture proportion.

Pool	β_YRI_(s.e.)	β_CEU_(s.e.)	β_CHB/JPT_(s.e.)	Intercept(s.e.)	N_SNP_	Method
MAY	0.824(0.0003)	0.175(0.0003)	n.d.	−0.00056(0.0001)	378,337	Pooling
	0.812(0.0002)	0.178(0.0002)	n.d.	0.00495(0.0001)	854,156	Genotype
	0.806(0.0003)	0.176(0.0003)	n.d.	0.00848(0.0001)	377,880	Genotype
	0.833	0.167	n.d.	n.a.	699	AIMs
GXE	0.868(0.0003)	0.122(0.0003)	n.d.	0.00434(0.0002)	353,260	Pooling
SPT	0.822(0.0004)	0.101(0.0004)	n.d.	0.0376(0.0002)	303,269	Pooling
MEC-AA	0.713(0.0003)	0.241(0.0003)	n.d.	0.0189(0.0001)	476,847	Pooling
MEC-H	0.056(0.0007)	0.319(0.0009)	0.599(0.0008)	0.0123(0.0003)	306,138	Pooling
	0.035	0.328	0.637	n.a.	69	AIMs
MEC-L	0.080(0.0006)	0.611(0.0008)	0.292(0.0007)	0.0122(0.0003)	358,822	Pooling
	0.052	0.660	0.288	n.a.	69	AIMs

The proportion of admixture for each of the admixed populations pooled in this study was estimated using a regression-based method (see Methods). Wherever possible, we also estimated the proportion of admixture using genotypes at AIMs known to distinguish the HapMap populations. β_YRI_, β_CEU_, and β_CHB/JPT_ are the regression coefficients, which are taken as the proportion of ancestry contributed by each of the YRI, CEU, and CHB/JPT populations. The standard error (s.e.) of the regression coefficient is also listed when available. Note that the s.e. may be biased downward, due to LD between SNPs. However, the s.e. estimates based on the LD-pruned set of SNPs are on the order of 10^−3^ (data not shown). Intercept is the regression intercept, which in this case is half of the unexplained ancestry in the model, as the average allele frequency for the population of interest and each of HapMap populations is ∼0.5 (Text S2). N_SNP_ is number of SNPs used to generate the ancestry estimates (see Methods). The “method” column indicates the method used to generate the admixture estimates: “pooling” indicates that estimates are based on regression from pooled allele frequencies, “genotype” indicates that estimates are based on regression from individual genotype data, and “AIMs” indicates that estimates are were generated using STRUCTURE and individual genotype data from a small number of AIMs (see Text S1). n.d. denotes not determined; n.a. denotes not available. Estimates based on the regression approach do not appear to be confounded by issues due to collinearity (data not shown). For MAY, GXE, and SPT, when SNP allele frequencies from CHB/JPT were included in the model, β_YRI_ was be largely unchanged, but a small contribution (<0.025) from CHB/JPT was estimated. This small admixture contribution from CHB/JPT appears to be largely an artifact due to sampling variation of the CEU and CHB/JPT reference populations (C.W.K.C., unpublished).

To extend this method to additional admixed populations, we applied our regression method to the MEC-H and MEC-L pools, using allele frequencies in all three HapMap populations as the predictor variables. We estimated the Native Hawaiians to be closest to ∼5.6% YRI, ∼31.9% CEU, and ∼59.9% CHB/JPT, and the Latinas to be closest to ∼8.0% YRI, ∼61.1% CEU, and ∼29.2% CHB/JPT (Table 1). These estimates are consistent with our knowledge of the population history for Native Hawaiians and Latinas (as East Asians are useful, though imperfect, surrogates for the ancestral Native American and Polynesian populations due to their relatively recent divergence from East Asians [11],[35],[36]), and are again very close to STRUCTURE-generated estimates based on 69 published AIMs previously typed in the MEC-H and MEC-L populations (∼3.5% YRI, ∼32.8% CEU, ∼63.7% CHB/JPT for MEC-H; ∼5.2% YRI, ∼66.0% CEU, ∼28.8% CHB/JPT for MEC-L, Table 1) [15]. We further estimated the MEC-AA pools to most closely correspond to 71.3% YRI and 24.1% CEU, the GXE pools to correspond to ∼86.8% YRI and ∼12.2% CEU, and the SPT pools to correspond to ∼82.2% YRI and ∼10.1% CEU (Table 1). Qualitatively, these estimates are consistent with reported estimates based on populations of similar demographic history. Namely, the Jamaican samples are expected to have proportionally more African ancestry than African Americans from Illinois [33], while African Americans from Los Angeles, CA, are expected to have proportionally more European ancestry [37]. Interestingly, the SPT panel appears to have a component of missing ancestry (summed proportion of admixture  = 92.3%, Table 1, and not improved substantially when the JPT/CHB panel was included, data not shown), yet displays relatively low F_ST_ when compared to its pseudopopulation (Table S1; also see Discussion).

### Identification and validation of ancestry informative markers

**Figure 2:**
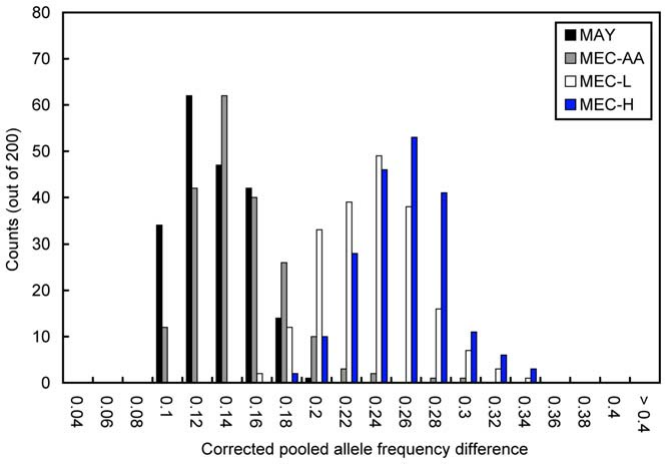
Distribution of allele frequency differences among the top 200 AIMs. The distribution of the corrected allele frequency differences between the estimated pooled allele frequency and that expected based on each population's respective pseudopopulation among the top 200 putative AIMs is shown for the MEC-AA, MEC-H, and MEC-L pools. Corrected pooled AF difference was calculated by fixing the AF in the pseudopopulation, computing the pooled AF in the appropriate direction given the deflated χ^2^ statistic, and then taking the difference. The distribution observed in the MAY pool represents the null distribution in which few additional validated AIMs are expected. To provide an estimate of the expected AF difference in a scenario where only sampling variation is responsible for the allele frequency difference between a population and its pseudopopulation, we simulated genotypes at ∼382 K SNPs for 521 individuals (the same number of post-QC SNPs and individuals as used in the MAY pools), drawing from the allele frequency in YRI 82% of the time and CEU 18% of the time, and compared the allele frequency of the simulated genotypes to that expected based on a 82%–18% mix of YRI and CEU. From this comparison, the top “AIMs” would only have an allele frequency difference of < ∼0.08.

To identify additional components of ancestry beyond those already modeled by the HapMap reference panels, we first constructed a corresponding pseudopopulation using the estimated admixture proportions for each of the populations pooled in this study. We then sought to identify potential AIMs that showed large differences in AF when comparing the pooled estimates to those based on the pseudopopulation (see Methods for details). To obtain an initial approximation of the number of AIMs expected, we examined the distribution of AF differences between the pooled population and its respective pseudopopulation among the top 200 AIMs (Figure 2, Figure S2). The distribution from the MAY pools serves as a null distribution for which few true AIMs are expected, as the admixture in this population is known to be very well described by the HapMap populations (F_ST_ = 0.0016 between the MAY pools and their pseudopopulation, Table S1). Relative to the distribution observed in the MAY pools, the distribution of the MEC-H pool displayed the most dramatic shift, followed by that of the MEC-L pool (Figure 2). The rightward shifts observed in the MEC-H and MEC-L pools are unlikely to be due to systematic error because the distribution of the MEC-AA pool (which was constructed and processed at the same time) appears similar to that observed in the MAY pools (Figure 2). On the other hand, the distributions from the GXE and SPT pools were similar in shape to that of the MAY pools, with only a slight rightward shift observed with the SPT pools (Figure S2). The relative degrees of rightward shift of the AF difference distributions corresponded with the rank order of the F_ST_ between the pooled panel and its respective pseudopopulation in all cases (Table S1), suggesting that the AIMs identified here are representative of the overall differentiation of the pooled panel and its pseudopopulation rather than being a biased set of SNPs that happen to show large AF differences due to pooling error. Taken together, these results suggest that many more AIMs with large AF differences informative for ancestral components not captured by the three HapMap panels likely exist in the MEC-H and MEC-L pools than in the MEC-AA and the Jamaican pools and can be identified through pooling.

**Figure 3:**
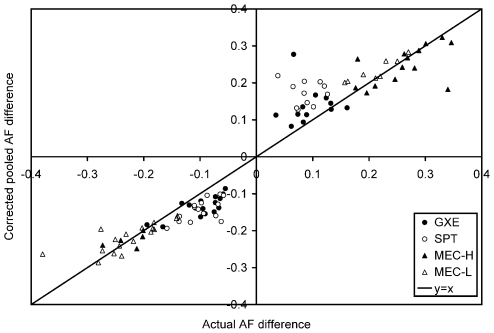
Validation by individual genotyping of the top putative AIMs in the individuals that comprised the pools. The actual AF difference between the population AF and that of the pseudopopulation was plotted against the corrected AF difference predicted by pooling for 25, 28, 26, and 19 of the top candidate AIMs in MEC-L, GXE, SPT, and MEC-H, respectively. Corrected pooled AF difference was calculated as in Figure 2. Filled circles represent results from GXE, unfilled circles are those from SPT, filled triangles are those from MEC-H, and unfilled triangles are those from MEC-L. In all three populations the classification of a putative AIM as either “encouraging” or “inconclusive” (see Methods) did not appear to correlate with the probability of successful validation (data not shown).

To validate the putative AIMs identified by pooled genotyping, we successfully genotyped 25, 28 and 26 of the top candidate AIMs in the individuals that comprised the MEC-L, GXE and SPT pools. For MEC-H, we examined 19 of the top 4000 AIMs (prior to pruning by distance) that had been already genotyped in the laboratory. Given the success of genotyping pooled DNA in identifying disease variants with large AF differences between cases and controls (see Introduction), we expected that the majority of the AIMs identified in the MEC-H and MEC-L panels would display true large AF differences between the pooled individuals and their corresponding pseudopopulations. Indeed, our estimates of AF differences in the MEC-H and MEC-L pools were generally quite close to the actual AF differences (Figure 3). A list of 438 and 431 putative AIMs genome-wide identified from MEC-H and MEC-L pools, respectively, is provided in Table S2. However, we tended to over-estimate the AF differences of the putative AIMs in the GXE and SPT pools (Figure 3), both of which have much lower F_ST_ values when compared to their respective pseudopopulations.

**Figure 4:**
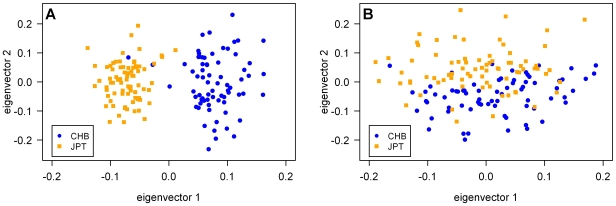
The top two axes of variation from principal component analysis of JPT and CHB. Results from EIGENSTRAT were based on (A) 420 putative AIMs selected by comparing the MEC-J pools to the CHD population from HapMap phase 3 and (B) 420 random SNPs. Differentiation between JPT and CHB is clear when using the set of putative AIMs, compared to that using the same number of random SNPs. Note that the two CHB individuals within the JPT cluster in (A) would also cluster with JPT individuals if genome-wide data were used (data not shown). Similar differentiation using random SNPs could also be achieved when ∼3,100 random SNPs were used (data not shown).

To further demonstrate that the AIMs selected via pooling would be informative in differentiating closely related populations, we sought to identify AIMs informative for distinguishing the two East Asian HapMap panels often grouped together by investigators: JPT (Japanese) and CHB (Han Chinese) (F_ST_ = 0.0067). We first removed population outliers along any of the top 10 principal components by EIGENSTRAT [4] using genome-wide Affymetrix 6.0 genotypes from HapMap phase 3 for JPT, CHB, and CHD (Chinese from Metropolitan Denver, Colorado). Using genome-wide data, JPT was clearly distinguishable from the two Chinese populations along the first axis of variation (eigenvector 1), with the second axis (eigenvector 2) starting to separate CHB from CHD, possibly reflecting a north to south cline among the Chinese (data not shown). We identified AIMs by comparing the MEC-J pools to CHD (which are both composed of Asian American individuals), and tested whether the 420 putative AIMs would be able to distinguish JPT from CHB. Indeed, using the panel of 420 putative AIMs, JPT and CHB were clearly separated from each other along the top principal component (Figure 4A). Based on this set of AIMs, the F_ST_ between JPT and CHB is 0.026, with a correlation of 0.946 with the true axis of variation (inferred by genome-wide data; discussed in [8],[14]). A set of 420 random SNPs was not able to distinguish the two East Asian populations (Figure 4B); ∼3100 random SNPs were necessary to achieve the same level of correlation with the true axis of variation (data not shown). Thus, AIMs identified via pooling should be informative for distinguishing even two relatively closely related populations (*e.g.*, JPT and CHB), and will likely be effective in distinguishing populations from neighboring countries (*e.g.*, divergent European populations, where F_ST_ is typically on the order of 0.01 [4]).

Overall, these results support our hypothesis that pooled genotyping may be most effective for detecting variants with large AF differences and that more AIMs exist in our Native Hawaiian and Latina cohorts that remain to be discovered. Additionally, this also suggests that the HapMap populations model the true genetic ancestry for the Jamaican populations accurately enough such that few SNPs with large AF differences would be detected.

## Discussion

Genotyping of pooled DNA has previously been proposed to be useful for several purposes. First, it has been shown that GWA studies using pooled DNA can efficiently screen large cohorts for variants with large AF differences between cases and controls [20], [27]–[30]. Second, it has been shown that the ability to resolve individuals contributing trace amounts of DNA to a pool holds great promise for forensic science [38]. Here we have proposed and demonstrated that genotyping of pooled DNA using genome-wide arrays is an efficient means to identify AIMs and to estimate global ancestry.

As the first study evaluating the efficacy of genotyping pooled DNA on the Affymetrix 6.0 platform, we first established a set of four SNP QC filters and showed that together the filters eliminated the vast majority of SNPs falsely displaying large allele frequency differences between pools (Figure 1), although at the apparent cost of an increased false negative rate (see Methods, and data not shown). Using SNPs that passed our stringent QC filters, we demonstrated that the estimated admixture proportions for our admixed panels were very similar to those obtained using current techniques and were robust to any remaining pooling-specific error (Table 1). Note that while we adopted a linear regression approach to estimate admixture proportions, variable transformations (such as the logit-transformation) or other forms of regression analysis for modeling rates and proportions could also be considered.

For the MEC-H and MEC-L panels, whose genetic ancestries were not sufficiently modeled by HapMap reference panels, we identified hundreds of AIMs with large AF differences by comparing these panels to their respective pseudopopulations and validated the top AIMs by individual genotyping (Figure 3). As markers informative for ancestry are those displaying large AF differences between populations (in this case, ∼20% difference in the MEC-H and MEC-L pools), our successful identification of AIMs is consistent with the reported identification of disease variants with large AF differences in case-control studies using pooled DNA [20], [27]–[30]. For identifying markers with moderate AF differences (in this case, ∼10% difference in the GXE and SPT pools), pooling tends to overestimate the differences (which is expected due to the “winner's curse”) but can still identify such SNPs (Figure 3). We also showed that AIMs identified via pooling are effective in differentiating the two East Asian HapMap populations (CHB and JPT) using principal components analysis (Figure 4).

In contrast to the MEC-H and MEC-L panels, the Jamaican pools (GXE and SPT) appeared to be much better modeled using just the YRI and CEU reference panels when we compared the distribution of AF differences among the top putative AIMs (Figure 2) and the estimated F_ST_ between the pooled sample and its pseudopopulation (Table S1), to those from the African American (MAY) pools. As a result, we anticipated and determined that most AIMs identified in the Jamaican pools have AF differences with moderate values from ∼8% to 15%. Moreover, we noted that the SPT pools appeared to have a missing component of ancestry unexplained by the HapMap YRI and CEU panels (summed proportion of admixture  = 92.3%, Table 1, and not improved substantially when the JPT/CHB panel was included, data not shown). The AIMs identified by comparing SPT to its pseudopopulation should be indicative of the missing ancestry. Given the modest AF differences detected between SPT and its pseudopopulation, it appears that these AIMs are informative for a between-population difference less than that expected for a between-population difference across continents (data not shown). Therefore, we suspect that the missing ancestry is from a population more similar to either the YRI or the CEU panel (or that YRI and/or CEU are inappropriate populations to serve as the ancestral populations for SPT), rather than due to contributions from other continental populations. Although it may appear contradictory that many more AIMs with large AF differences were detected in the MEC-H and MEC-L pools, despite a much higher level of genetic ancestry explained (summed proportion of admixture  = 97%–98% using all three HapMap panels) than the Jamaican pools, this likely reflects the fact that the HapMap East Asian panels are acceptable, but not perfect, proxies for Polynesian or Native American ancestries on average. Thus, at least a subset of the AIMs identified in MEC-H and MEC-L should be informative for the difference between East Asians and Polynesians or Native Americans (*e.g.*, due to drift). Therefore, the extent of the summed proportion of admixture of a pooled panel will not necessarily correlate with the expected number of AIMs with large AF differences.

In light of the results presented here, we envision that studies using pooled DNA have great potential utility for future association studies. Given the success of identifying variants with large effect sizes using pooled DNA [20], [27]–[30], one potential use of genotyping pooled DNA is to quickly screen for the presence of variants with large effect sizes, which can provide guidance to study design for additional GWA studies using individual DNA. Moreover, we have shown that studying pooled DNA can be used to evaluate genetic ancestry and potential population substructure in the context of association studies. As future association studies expand beyond populations of European ancestry, our approach should allow rapid assessment of global ancestry to identify AIMs. Once AIMs are validated and genotyped in the study population, individual level genetic ancestry as well as local ancestry can be estimated for use as covariates in association studies where genome-wide data are not available. As genome sequencing and SNP discovery projects for additional species are completed, pooling-based experiments may also be an efficient first step in assessing genetic structure in populations from other species. Lastly, a rigorous assessment of GWA studies using pooled DNA for identifying disease variants with small effect sizes is needed. Our African American and Jamaican samples here were initially pooled by thresholded BMI, and the MEC samples were pooled by age at menarche status (see Text S1). A preliminary attempt to identify variants associated with BMI or age at menarche showed enrichment of variants with nominal associations when genotyped individually (C.W.K.C., Z.K.Z.G., J.N.H., unpublished). However, our power to detect strongly associated variants may have been limited by the number of replicates genotyped to control for error due to pooling, limitations of the platform used, and the small sample size relative to the expected effect sizes, and thus was not a focus of this paper.

Although we utilized the availability of individual genotypes in informing our QC filters, individual level genotypes are not required to establish filter parameters. Given a population of individuals randomly pooled into multiple pools in order to assess the genetic ancestry of the population, one can compare pools in a pair-wise case/control-like fashion where no associations would be expected. Then, by assessing changes in the genomic control inflation factor [5] when different QC filter cut-offs are applied, one can adjust the filter parameters to suit the goals of the study and to reflect varying levels of tolerance for false positives. Therefore, for the three of the four filters established here that do not depend on individual genotypes (FLD-filter, r-filter, and maf-filter), data quality and the study population will dictate the number of SNPs filtered given a particular threshold.

Finally, it should be noted that the recommendations for use of genotyping pooled DNA on a genome-wide array – to determine genetic ancestry, to screen for disease variants with large AF differences, and to study population demographics – are made based on the current state of the technology and methodology. Given our experience with the Affymetrix 6.0 platform, we have focused on applications that require the detection of moderate to large allele frequency differences. We anticipate that advances in the genotyping platform and improvements in sample handling may enhance the overall data quality and accuracy of allele frequency estimates, and that the same filter parameters may retain more SNPs for analysis than did the conservative approach taken here. Thus, given a sufficiently robust platform, it may be increasingly possible to efficiently search genome-wide for variants that have small allele frequency differences between samples using pooled DNA.

## Methods

### Study populations and DNA pool construction

The cohorts used in this study consisted of 775 African American individuals from Maywood, IL (MAY); 1,039 and 1,467 Jamaican individuals from Kingston (GXE) and Spanishtown (SPT), Jamaica, respectively; and women from the Hawai'i and Los Angeles Multi-Ethnic Cohort (MEC) [39]: 391 African Americans (MEC-AA), 298 Native Hawaiians (MEC-H), 363 Latin Americans (MEC-L), and 202 Japanese Americans (MEC-J). In total, we constructed 22 DNA pools from the individual DNA samples: four pools from 521 MAY individuals, six pools from 688 GXE individuals, four pools from 480 SPT individuals, and two pools each from 321 MEC-AA, 252 MEC-H, 332 MEC-L, and 202 MEC-J individuals. Pools were initially constructed in case/control fashion by dichotomized BMI and age at menarche status (Text S1). For the purpose of identifying AIMs in this study the pools differing in menarche or obesity status were treated as independent samples from their respective admixed populations.

### Pooled allele frequency estimation by polar transformation of raw data

The Birdseed algorithm [40] was used to estimate AA, AB, and BB cluster means and covariances of probe intensities for individuals on the same plate as the pooled samples, as well as to call the genotypes for these samples. Pooled samples were processed in the same fashion as individual samples, with the exception of using only median normalization without quantile normalization. Informed by the covariance matrices of the three genotype classes of the individuals on the plate, we calculated the angle *θ_AA_* measuring the degree of rotation of the AA genotype cluster with respect to the horizontal axis (*i.e.*, the probe intensity space of allele A) for each autosomal SNP as the following (Figure S3, Text S2):
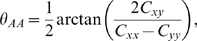
where *C_xy_*, *C_xx_*, and *C_yy_* are from the covariance matrix of the AA genotype cluster:
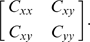

*θ_AB_* and *θ_BB_* were calculated similarly, using the appropriate covariance matrices. The intersection of the two lines angled at *θ_AA_* and *θ_BB_* and intersecting the center of the AA and BB genotype cluster centroids, respectively, was defined as the origin (*O*), with respect to which new axes x' and y' were established. We then defined *θ_pool_*, the angle of the replicate pool intensity with respect to the x' axis as:
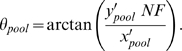

*x'_pool_* and *y'_pool_* represent the x'- and y'-coordinates of the replicate pool intensity, and NF is the normalization factor to adjust for differential allelic signal intensities using the location of the AB genotype cluster, akin to the various forms of *k*-correction proposed (for example, [41]), given by:
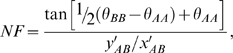
where *x'_AB_* and *y'_AB_* represent the x'- and y'-coordinates of the center of the AB genotype cluster.

To estimate the pooled allele frequency (AF) for the A allele for each replicate given *θ_pool_*, we used the following conversion:
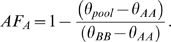
AF estimates for all replicates from a given pool were averaged to obtain the final pooled AF estimate.

### Quality control filters

Informed by the genotype data from the individuals comprising the MAY pool, we explored several possible filtering methods to identify those that most efficiently eliminated SNPs that genotyped inconsistently in pooled DNA. We first compared the distributions of the 200 worst and best performing SNPs with respect to parameters of various potential filters to determine both which filtering methods were most effective and to approximate values for filter cut-offs. The worst and best performing SNPs were defined as follows: for each SNP, we calculated the corrected χ^2^ test statistic ([21] and described below) by comparing the two case pools to the two control pools from the MAY panel (Text S1) (using both actual genotypes and pooled estimates of AF). The worst performing SNPs were defined as those with the greatest corrected χ^2^ difference between individual data and pooled data. The best performing SNPs were defined as those with the least χ^2^ difference among SNPs with the most significant χ^2^ test statistics. We then defined the proportion of false positives (PFP) as the proportion of SNPs with an expected (based on individual genotyping) *P*-value of >0.05 that were ranked among the top 0.05% SNPs by estimated pooling *P*-value. PFPs were calculated for the pre- and post-filtered list of SNPs at various filter cut-offs to establish the final values used for each filter.

In the manner described above, we established three filters that were effective in eliminating SNPs that genotyped poorly or inconsistently: 1) separation of individual genotype clusters based on Fisher's linear discriminant, a measure of distance between two clusters (FLD-filter), 2) radius of intensity of the signal from pooled DNA (r-filter), and 3) population minor allele frequency estimated from pooled DNA (MAF-filter) (see Text S1 and Figures S4, S5, and S6 for details). In all cases we strived for filter cut-offs that stringently eliminated poorly performing SNPs while retaining sufficient SNPs for broad coverage of the genome (Figure S4A, S4B and Figure S6). Applying these three filters left ∼382 K SNPs for association analysis within the MAY panel, comparing the case pools to the control pools. The QC filters lowered the PFP from 0.793 to 0.642, and improved the genomic control (GC) inflation factor [5] from 1.52 to 1.38. Among the 809 independent SNPs with a *P*-value of 0.001 or lower (based on individual genotyping), 397 SNPs (or at least one proxy with r^2^>0.8) passed the three QC filters in pooling, for a false negative rate of 0.509 due to QC filtering. (Note that post-QC SNPs are still subject to pooling-specific error, which is not yet accounted for at this step in the process.) The relatively elevated inflation factor after applying the three filters likely represents error in the pooled AF estimates we were unable to account for in our study design. As one is often searching for variants with small AF differences between case and control groups in a disease association, we also recommend fitting the distribution of the pooled AF estimates from the case and control pools to the overall pooled AF distribution to ensure a similar distribution of AF estimates between the case and control pools. In our experience this further lowers the inflation factor (from 1.38 to 1.08 in our data) and improves the PFP (C.W.K.C., unpublished).

By taking advantage of the individual genotypes from the MAY pools, we also established a filter to measure the consistency of the AF estimates for each SNP. Over the four MAY pools, we calculated the difference in the AF estimates between the pooled sample and the individual samples, and the variance across the four pools was used as a measure of consistency of the AF estimates (hist-filter, see Text S1 for details). The effectiveness of the cut-off values for this filter was established by the changes in the GC inflation factor of a presumed null distribution in the comparison of one of the MEC-AA pools to the other (Figure S4C). All four filters were applied in the analysis of all pooled panels other than the MAY panel in this study.

### Estimation of admixture proportion

To estimate the proportion of admixture in the pooled populations, we employed a linear regression model where the estimated allele frequency for SNP *i* was modeled as follows:
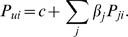

*P_ui_* is the estimated allele frequency from pooling in the population of unknown admixture for SNP *i*, and is regressed on independent variables *P_ji_*, which is the allele frequency in the ancestral (reference) population *j* for SNP *i* with respect to allele A according to the Affymetrix 6.0 array annotation (http://www.affymetrix.com/support/technical/annotationfilesmain.affx, GenomeWideSNP_6_Annotations, na25). *β_j_* is the regression coefficient and is an estimate of the proportion of contribution from population *j*, and *c* is the constant combining error and unexplained ancestry (*i.e.*, the intercept). Because allele A assignment on Affymetrix 6.0 array is independent of the minor allele at the locus, E(*P_ji_*) = E(*P_ui_*) = 0.5, which is necessary for the accurate estimation of *β_j_* using regression (Text S2). *β_j_*'s and their standard errors were estimated by multivariate linear regression using the method of least squares in R version 2.4.0 (Vienna, Austria; http://www.r-project.org/), using all SNPs that passed our QC filters (see above) and had genotyping success rates >0.8 in all three HapMap populations (YRI, Yoruba in Ibadan, Nigeria; CEU, Utah residents with ancestry from northern and western Europe; JPT/CHB, combined Japanese in Tokyo, Japan and Han Chinese in Beijing, China). We used the three HapMap populations genotyped on the Affymetrix 6.0 array as our reference ancestral populations [42]. For estimating admixture proportions in MAY and MEC-AA, YRI and CEU were used as the reference populations. For estimating admixture proportion in the remaining pools (GXE, SPT, MEC-H, and MEC-L), YRI, CEU, and combined JPT/CHB were used as the reference populations. For each pooled population, a corresponding pseudopopulation was constructed, in which the allele frequency of each SNP was calculated using the allele frequency catalogued in each of the reference populations, weighted by the estimated proportion of admixture, and adding the constant *c*.

### Deflation of the test statistic using pooled DNA

One factor that influences the analysis of pooled but not individual genotype data is that when DNA pools are genotyped, an estimated rather than observed number of allele counts is obtained. The variance around the estimated allele frequency obtained from pooled genotyping includes variance that arises specifically due to pooling in addition to the sampling variance. If the additional variance is not taken into account, a standard χ^2^ statistic will have a greatly inflated value. Here we corrected for this χ^2^ statistic inflation using a method proposed by Visscher and Le Hellard [21],[43], where the corrected statistic, 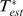, is given by:
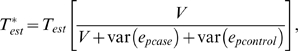
where 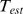 is the standard (naïve) χ^2^ statistic based on estimated allele counts derived from the estimated pooled allele frequency. (Note that when calculating 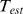, the minor allele frequency in either the case or the control pools must be >0, otherwise the χ^2^ statistic cannot be calculated. Thus while not a formal QC filter, any SNP in which the estimated minor allele frequency was <0 in either the case or the control pool, a situation that would arise for very rare SNPs or erroneous hybridization signals, was dropped from analysis.) *V* is the sum of the sampling variance for the case and control pools, given by:
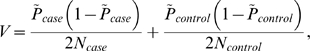
where 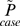 and 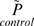 are the estimated pooled AF for the case and control pools, respectively. Var(*e_pcase_*) and var(*e_pcontrol_*) are the squared standard errors among the pooled AF estimates from all of the replicates for the case and control pools, respectively. *V*, var(*e_pcase_*) and var(*e_pcontrol_*) were calculated for each SNP tested for association. When multiple case or control pools were available, the total pooled allele frequency used was the weighted average (by number of individuals in the pool) of the pooled allele frequencies estimated for each pool. The pooled variance, var(*e_p_totcase_*), is then given by:
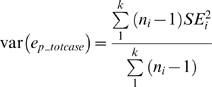
for *k* case pools each with *n_i_* replicates. *SE_i_* is the standard error of the estimated AF for the *i*th pool. The pooled variance for the control pool was calculated similarly.

When identifying AIMs informative for ancestry over and above that explained by available reference panels, all pools from the population being studied were designated the “case” pools, and the pseudopopulation was used as the “control” pool. In this case, the sampling variance for the pseudopopulation was based on a population size of either 120 individuals (if only YRI and CEU were used) or 210 individuals (if YRI, CEU, and JPT/CHB were all used). Pooling specific variance for the pseudopopulation was assumed to be 0.

### Identification of ancestry informative markers (AIMs)

Ancestry informative markers were selected for the GXE, SPT, MEC-H, and MEC-L panels by comparing the estimated allele frequency in each population by pooling to its respective weighted reference panel (pseudopopulation), or for the MEC-J panel by comparison to the AF from the HapMap phase 3 CHD (Chinese from Metropolitan Denver, Colorado) population (http://www.hapmap.org). Pseudopopulations were constructed based on the estimates of admixture proportion using the HapMap populations as proxies for the ancestral populations.

For the Jamaican pools, SNPs were divided into three categories, based on the *P*-values associated with the surrounding SNPs in linkage disequilibrium (LD) with the SNP of interest. Here, *P*-values measure the extent to which pooled allele frequencies differ from those expected using the pseudopopulation. LD was determined using the set of pre- and post-QC filtered sets of SNPs, based on the HapMap YRI population; SNPs within 20 Mb of the SNP of interest were considered to be in LD if they had r^2^>0.5 in HapMap YRI with the SNP of interest. “Encouraging” SNPs had at least one SNP in LD with a GC-corrected *P*-value<0.05 and had at least half of the surrounding SNPs (those in LD) with GC-corrected *P*-values<0.1. “Discouraging” SNPs had none of the SNPs in LD with GC-corrected *P*-values<0.1. The remaining SNPs were categorized as “inconclusive,” a category also encompassing SNPs with no other SNPs in LD. Non-discouraging (*i.e.*, encouraging or inconclusive) AIMs were then further pruned to remove any AIMs within 4 Mb of each other to obtain a panel of independent AIMs.

We chose a set of 50 candidate AIMs each in GXE and SPT to be validated by individual genotyping, using two complementary approaches. First, we selected the top 25 SNPs based on GC-corrected *P*-value, excluding any SNPs categorized as discouraging when either the filtered or unfiltered set of SNPs in LD was examined for categorization. Second, we selected an additional 25 SNPs with GC-corrected *P*-values <1×10^−3^, at least 2 SNPs in LD with the SNP of interest from the unfiltered dataset, and a categorization of encouraging when using both the filtered and unfiltered datasets for a set of SNPs in LD. For this second list, we chose the SNPs with the largest number of SNPs that were in LD that also had *P*-values <0.05.

Identification of AIMs in the MEC-H, MEC-L, and MEC-J panels was performed similarly, with the exception that AIMs in MEC-H were not pruned by distance in order to allow validation using SNPs previously genotyped in those samples. The HapMap reference panel representing the major ancestry in each of the MEC pools was used as the reference panel for LD determination (*i.e.,* JPT/CHB for MEC-H and MEC-J, and CEU for MEC-L).

### Technical validation by individual genotyping

Predicted AIMs and obesity-associated SNPs were validated by individual genotyping in the individuals comprising the pools using the Sequenom MassArray system (see Text S1).

## Supporting Information

Figure S1 Estimated AF from pooling versus AF from individual genotyping, before and after QC filtering, of the MAY panel. Samples from the MAY panel were also genotyped individually, allowing us to plot the population allele frequency of the individuals that comprised the MAY pools against the estimated allele frequency as determined by pooled genotyping to examine the accuracy of allele frequency estimation using pooled DNA. The left panel includes ∼855 K autosomal SNPs for which individual genotype data exist prior to applying the SNP QC filters; the right panel includes ∼382 K SNPs after applying three of the four QC filters (see Methods, Text S1). (The hist-filter was not applied as it is reliant on the individual and pooled genotyping results from the MAY pool.) This comparison is based only on the average of the allele frequency estimates, without taking into account the error involved in such estimates, which is adjusted when calculating the association χ^2^ statistic (see Methods).
(0.23 MB TIF)

Figure S2 Distribution of allele frequency differences among the top 200 AIMs for the GXE and SPT pools. The top 200 putative AIMs in the GXE and SPT pools were identified as described in the text and in Figure 2. The distribution of allele frequency differences due solely to sampling variation is < ∼0.08, as discussed in the legend of Figure 2. For both panels the distribution appears similar to that of MAY, with a slight rightward shift seen in SPT only, suggesting that a weighted reference panel from the HapMap explains the majority of the genetic ancestry in these two Jamaican samples.
(0.21 MB TIF)

Figure S3 Origin of SNP intensity space and polar transformation of raw Affymetrix data. Genotypes for each individual on a given genotyping plate cluster into three genotype classes when plotting intensity of the A probe versus that of the B probe. By taking into account the covariance of the two intensities for the two homozygous genotype classes, the origin, *O*, is defined, conceptually, as the intersection between the two lines that run through the center of the two homozygous clusters angled in the same direction as the clusters (see Methods). Once the origin is defined, *θ_AA_* and *θ_BB_* can be determined and *θ_pool_* can be estimated for the pooled sample, which is then converted into the estimated allele frequency (see Methods). The red circles represent centers of genotype clusters; the blue circle represents the raw intensity of one replicate of a pooled sample.
(0.55 MB TIF)

Figure S4 Proportion of false positives and genomic coverage at various SNP QC filter cut-offs. The various cut-off values for the: (A) FLD-filter, (B) MAF-filter, and (C) hist-filter are plotted against the number of SNPs remaining after filtering and the effectiveness of the QC filter, as measured by proportion of false positives (PFP) in A and B, or by genomic control inflation factor in C. The solid lines correspond to the number of SNPs that passed the QC filter in the MAY pools (A and B) and in the MEC-AA pool (C), while the dotted lines correspond to the PFP. PFP is defined as the proportion of SNPs with an expected (based on individual genotyping) *P*-value of >0.05 ranked among the top 0.05% SNPs by estimated pooling *P*-value (after deflation of the χ^2^ statistic (see Methods)). For the FLD- and MAF-filters, a SNP passed the QC filter if its value was greater than or equal to the cut-off value; for the hist-filter, a SNP passed the QC filter if its value was less than or equal to the cut-off value. In all cases, more stringent cut-off values appeared to improve the PFP or the genomic control inflation factor, but decreased overall genomic coverage.
(0.33 MB TIF)

Figure S5 Determination of the *r*/*r'* ratio. We calculated the radius *r* as the distance from the origin *O* (calculated as described in Figure S1) to the raw chip intensity *P* (blue circle). *r* was then normalized using the expected value for an average individual DNA sample on the same plate, given by *r'*. *r'* was defined as the distance from the origin *O* to *I*, the expected intensity signal of the individual DNA sample given the same estimated allele frequency. There is one *r*/*r'* ratio for each pool replicate at each SNP. Red circles represent the centers of the genotype clusters.
(0.60 MB TIF)

Figure S6 Proportion of false positives and genomic coverage at various r-filter cut-offs. We evaluated the effect of using various cut-offs for the r-filter and requiring SNPs from a variable number of replicates (cases and controls combined) to pass the filter. Dark blue lines required all 13 case and control replicates of the MAY pools to have an *r*/*r'* ratio greater than or equal to the cut-off value to be retained for downstream analysis; light blue lines required 12 of 13 replicates; dark green lines required 11 of 13 replicates; light green lines required 10 of 13. Solid lines correspond to the number of SNPs passing the QC filter at the particular cut-off value; dotted lines correspond to the PFP. For all values of the filter cut-off, requiring fewer replicates to pass retained a greater number of SNPs. At cut-off values of 0.9 to 0.95 the PFP increased, perhaps reflecting the removal of real associations. To optimize both PFP and SNP coverage, either a cut-off value of 0.8, requiring 11 of 13 passing replicates, or a cut-off value of 0.85, requiring 12 of 13 passing replicates, may be appropriate. Here we adopted a cut-off threshold of 0.8 and required a pass rate of 80% among the replicates.
(0.64 MB TIF)

Table S1 F_ST_ between pooled panels and their respective pseudopopulations.
(0.03 MB DOC)

Table S2 List of ancestry informative markers identified from pooled Native Hawaiian and pooled Latina populations.
(0.09 MB XLS)

Text S1 Supplemental methods.
(0.07 MB DOC)

Text S2 Formula derivations.
(0.06 MB DOC)
