# Supplementary figures and images for: Rapid Assessment of Genetic Ancestry in Populations of Unknown Origin by Genome-Wide Genotyping of Pooled Samples
Source: PLoS Genet. 2010 Mar 5;6(3):e1000866. doi: 10.1371/journal.pgen.1000866 (PMC2832667; doi:10.1371/journal.pgen.1000866)

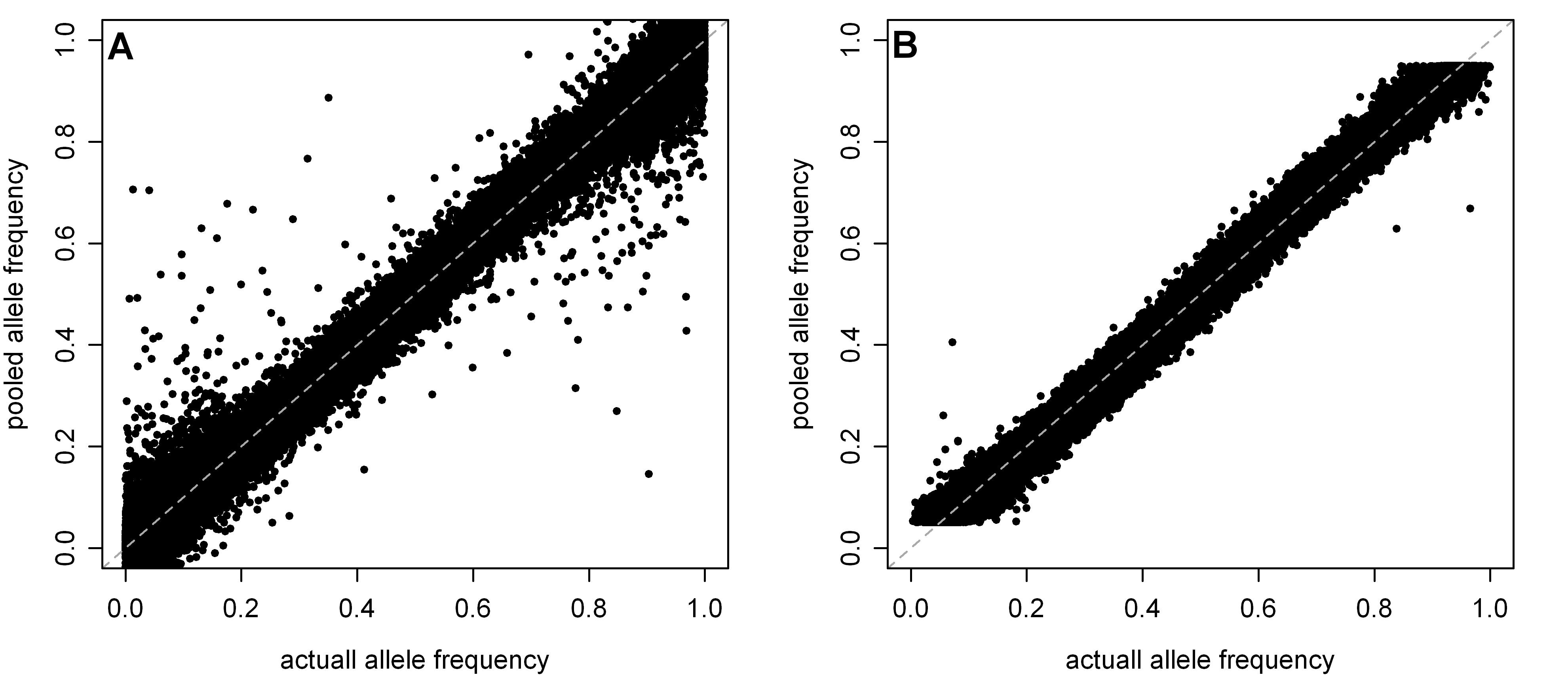

Supplement: Figure S1 — Estimated AF from pooling versus AF from individual genotyping, before and after QC filtering, of the MAY panel. Samples from the MAY panel were also genotyped individually, allowing us to plot the population allele frequency of the individuals that comprised the MAY pools against the estimated allele frequency as determined by pooled genotyping to examine the accuracy of allele frequency estimation using pooled DNA. The left panel includes ∼855 K autosomal SNPs for which individual genotype data exist prior to applying the SNP QC filters; the right panel includes ∼382 K SNPs after applying three of the four QC filters (see Methods, Text S1). (The hist-filter was not applied as it is reliant on the individual and pooled genotyping results from the MAY pool.) This comparison is based only on the average of the allele frequency estimates, without taking into account the error involved in such estimates, which is adjusted when calculating the association χ2 statistic (see Methods). (0.23 MB TIF) [file pgen.1000866.s001.tif]

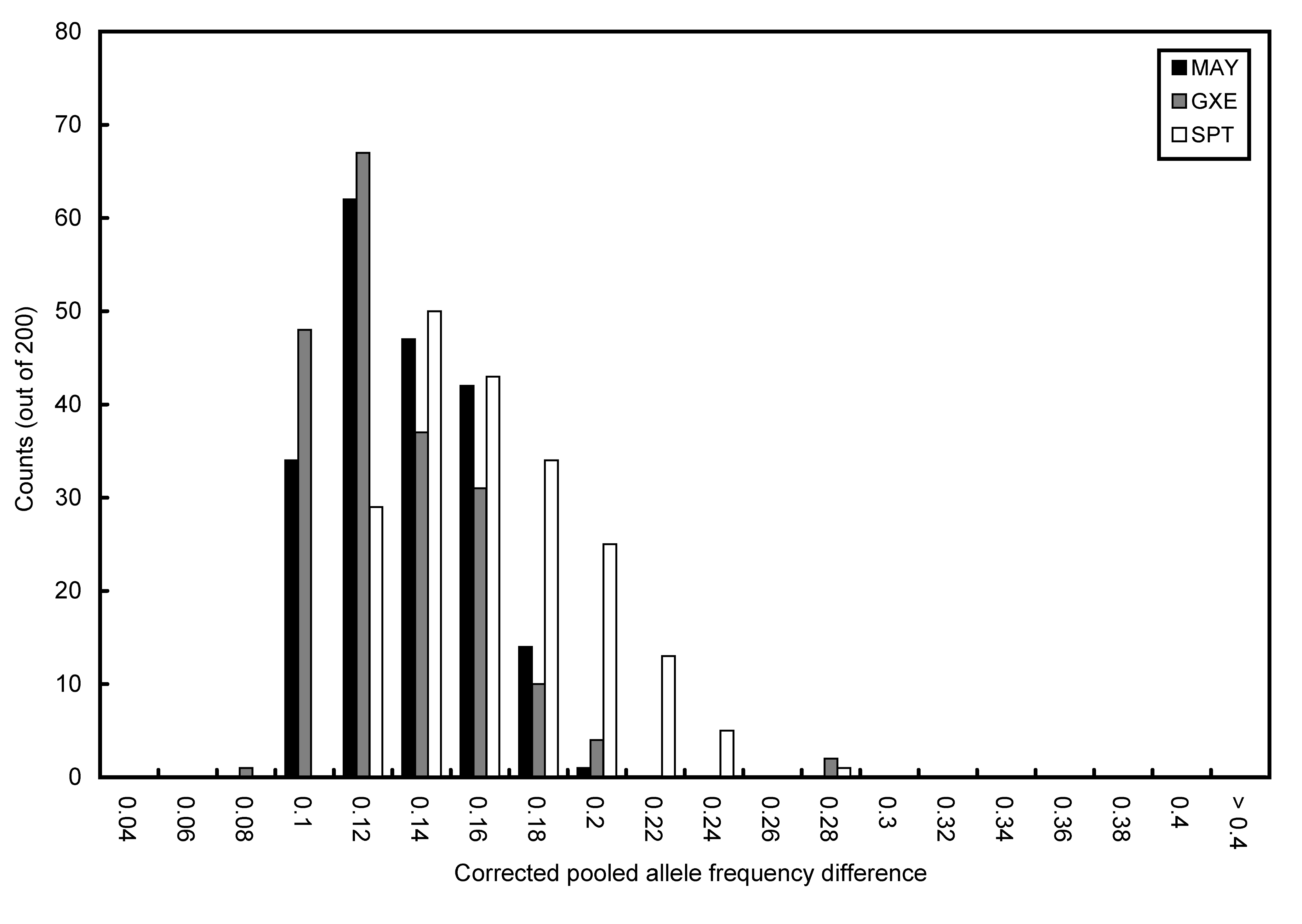

Supplement: Figure S2 — Distribution of allele frequency differences among the top 200 AIMs for the GXE and SPT pools. The top 200 putative AIMs in the GXE and SPT pools were identified as described in the text and in Figure 2. The distribution of allele frequency differences due solely to sampling variation is < ∼0.08, as discussed in the legend of Figure 2. For both panels the distribution appears similar to that of MAY, with a slight rightward shift seen in SPT only, suggesting that a weighted reference panel from the HapMap explains the majority of the genetic ancestry in these two Jamaican samples. (0.21 MB TIF) [file pgen.1000866.s002.tif]

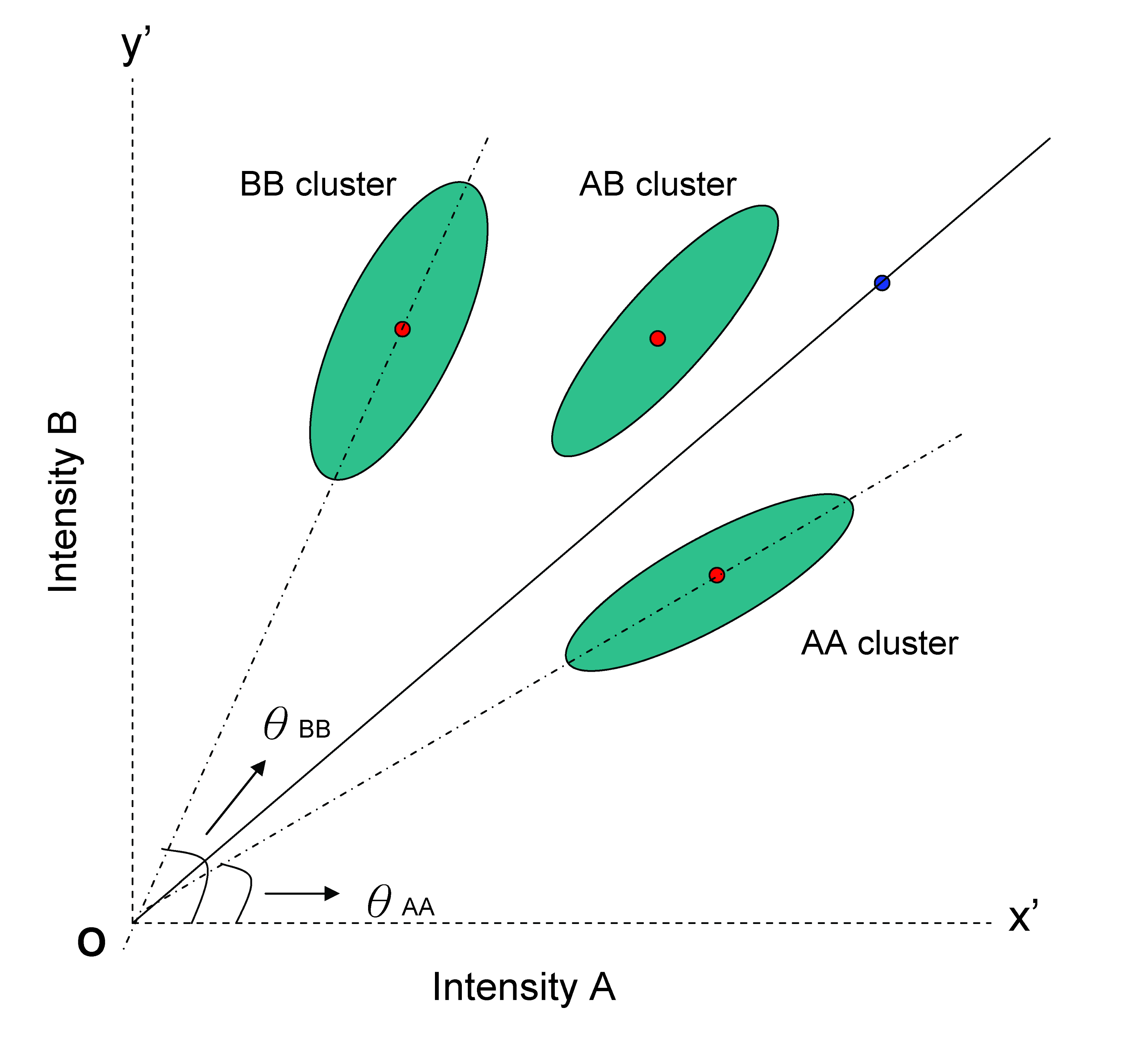

Supplement: Figure S3 — Origin of SNP intensity space and polar transformation of raw Affymetrix data. Genotypes for each individual on a given genotyping plate cluster into three genotype classes when plotting intensity of the A probe versus that of the B probe. By taking into account the covariance of the two intensities for the two homozygous genotype classes, the origin, O, is defined, conceptually, as the intersection between the two lines that run through the center of the two homozygous clusters angled in the same direction as the clusters (see Methods). Once the origin is defined, θAA and θBB can be determined and θpool can be estimated for the pooled sample, which is then converted into the estimated allele frequency (see Methods). The red circles represent centers of genotype clusters; the blue circle represents the raw intensity of one replicate of a pooled sample. (0.55 MB TIF) [file pgen.1000866.s003.tif]

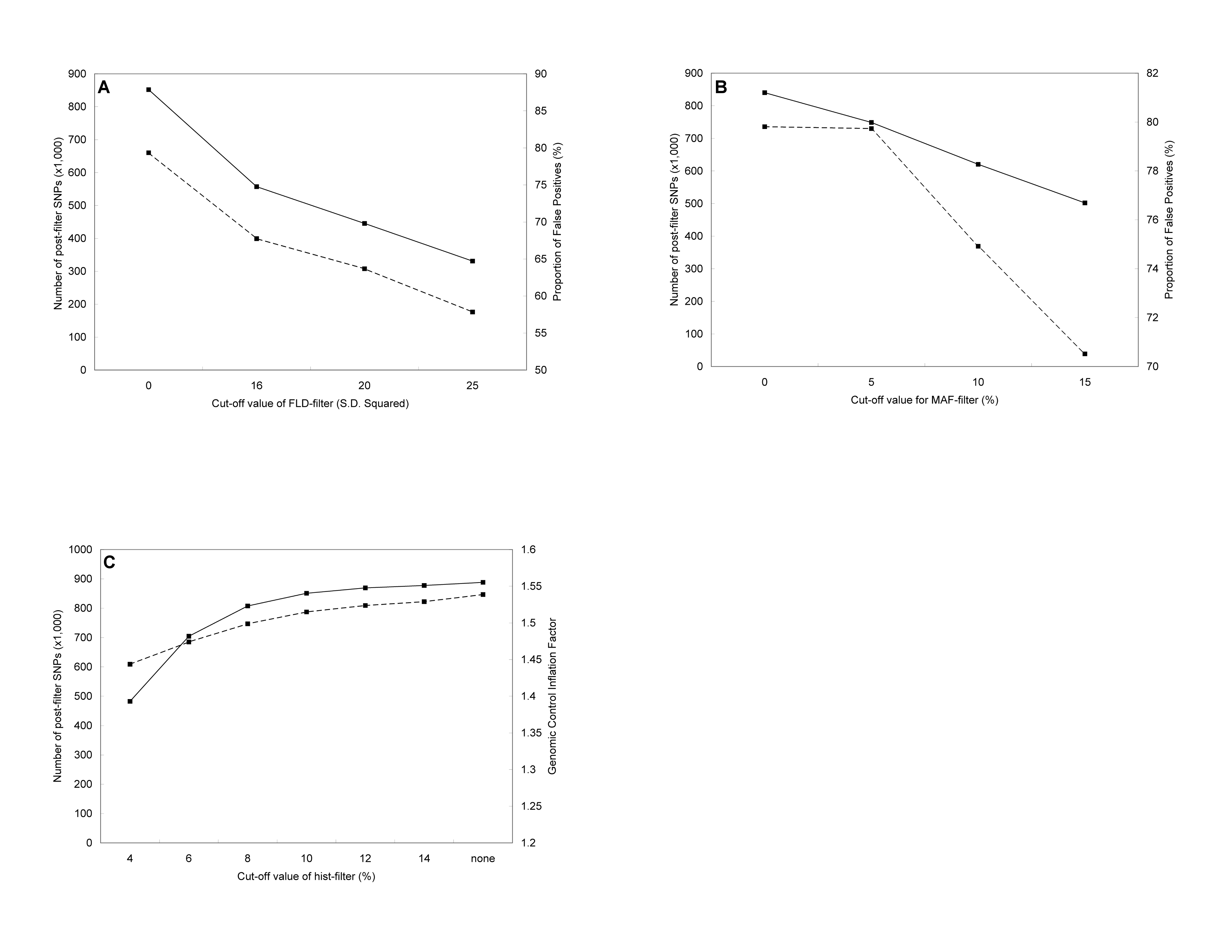

Supplement: Figure S4 — Proportion of false positives and genomic coverage at various SNP QC filter cut-offs. The various cut-off values for the: (A) FLD-filter, (B) MAF-filter, and (C) hist-filter are plotted against the number of SNPs remaining after filtering and the effectiveness of the QC filter, as measured by proportion of false positives (PFP) in A and B, or by genomic control inflation factor in C. The solid lines correspond to the number of SNPs that passed the QC filter in the MAY pools (A and B) and in the MEC-AA pool (C), while the dotted lines correspond to the PFP. PFP is defined as the proportion of SNPs with an expected (based on individual genotyping) P-value of >0.05 ranked among the top 0.05% SNPs by estimated pooling P-value (after deflation of the χ2 statistic (see Methods)). For the FLD- and MAF-filters, a SNP passed the QC filter if its value was greater than or equal to the cut-off value; for the hist-filter, a SNP passed the QC filter if its value was less than or equal to the cut-off value. In all cases, more stringent cut-off values appeared to improve the PFP or the genomic control inflation factor, but decreased overall genomic coverage. (0.33 MB TIF) [file pgen.1000866.s004.tif]

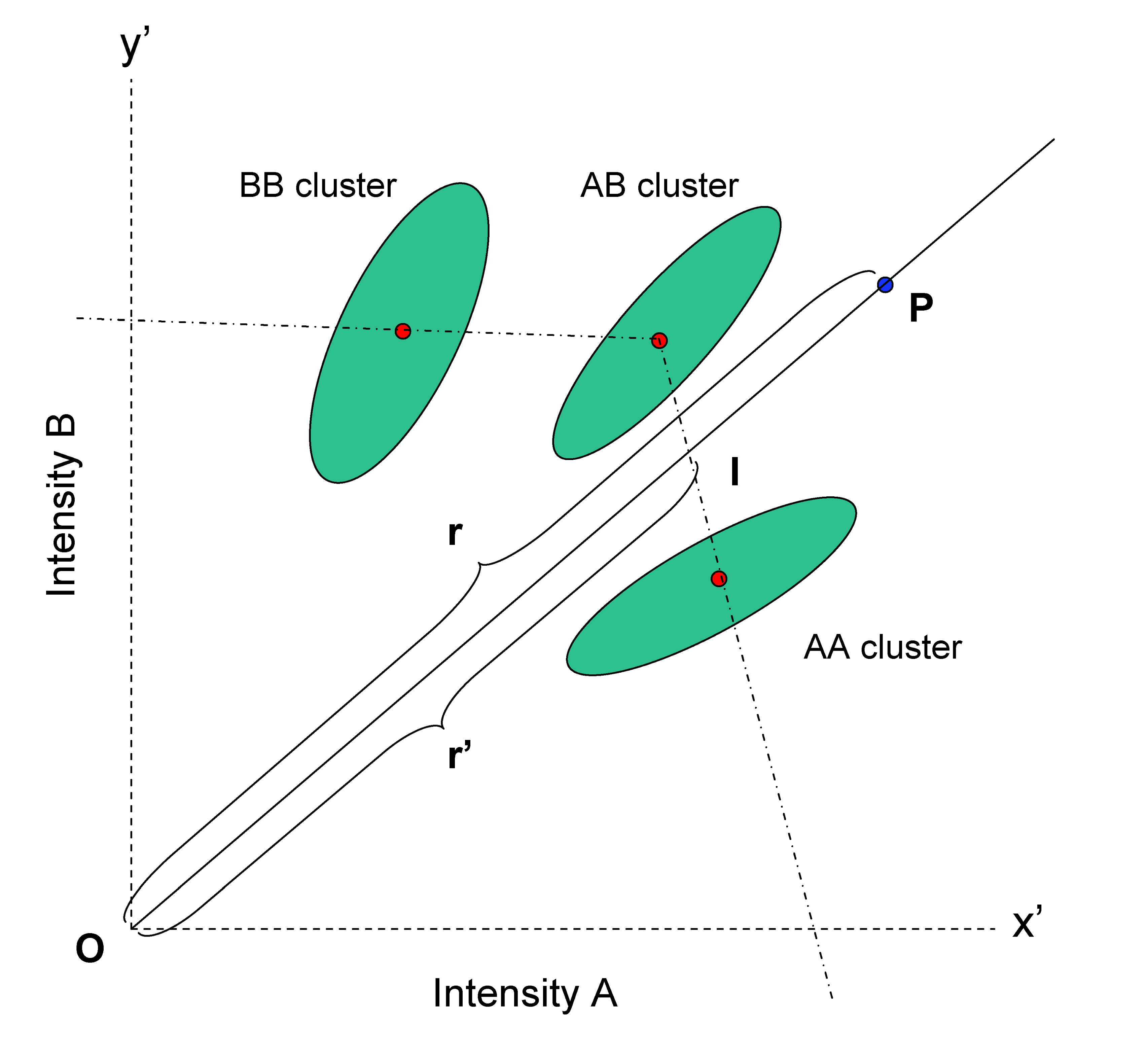

Supplement: Figure S5 — Determination of the r/r' ratio. We calculated the radius r as the distance from the origin O (calculated as described in Figure S1) to the raw chip intensity P (blue circle). r was then normalized using the expected value for an average individual DNA sample on the same plate, given by r'. r' was defined as the distance from the origin O to I, the expected intensity signal of the individual DNA sample given the same estimated allele frequency. There is one r/r' ratio for each pool replicate at each SNP. Red circles represent the centers of the genotype clusters. (0.60 MB TIF) [file pgen.1000866.s005.tif]

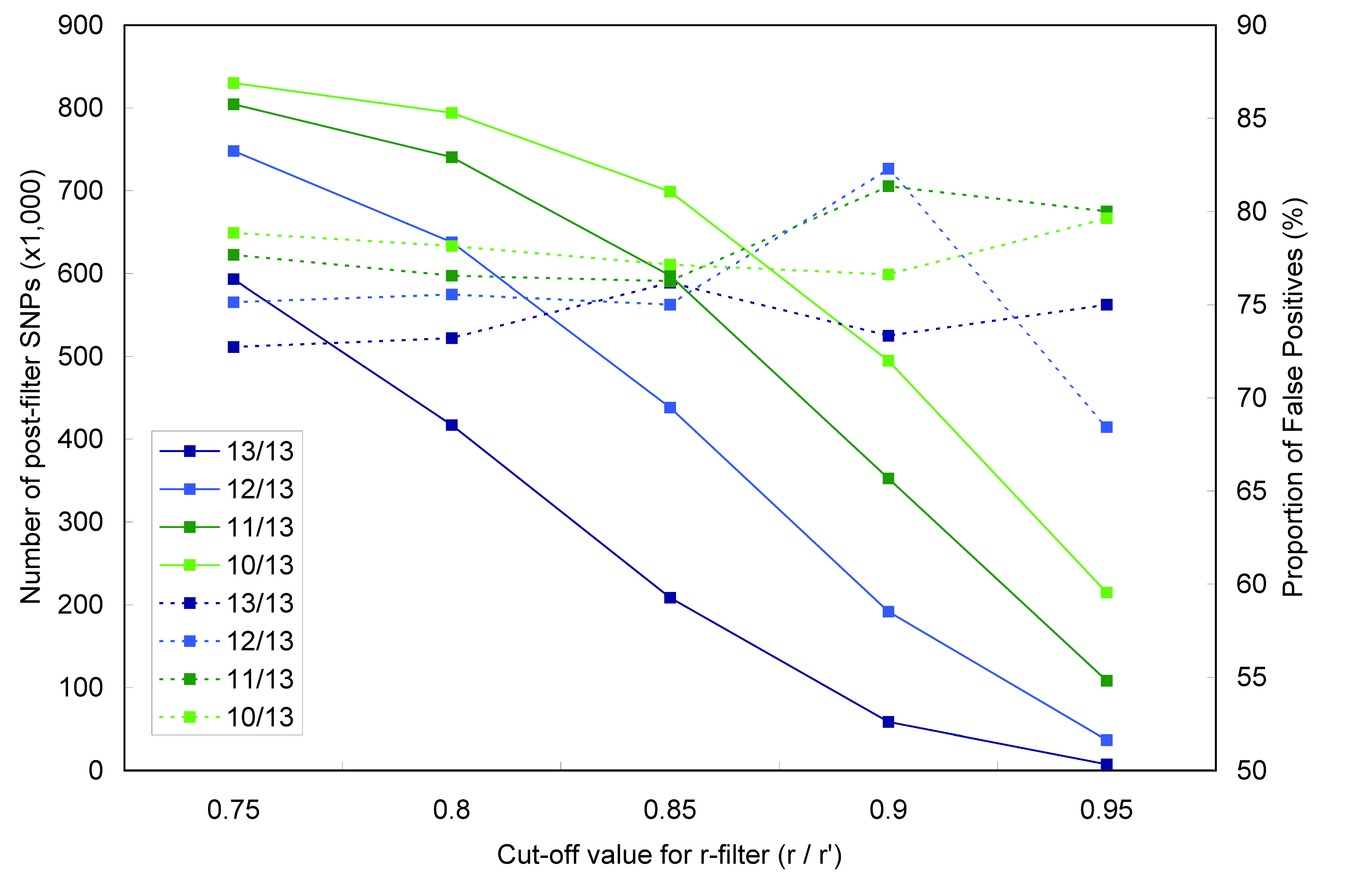

Supplement: Figure S6 — Proportion of false positives and genomic coverage at various r-filter cut-offs. We evaluated the effect of using various cut-offs for the r-filter and requiring SNPs from a variable number of replicates (cases and controls combined) to pass the filter. Dark blue lines required all 13 case and control replicates of the MAY pools to have an r/r' ratio greater than or equal to the cut-off value to be retained for downstream analysis; light blue lines required 12 of 13 replicates; dark green lines required 11 of 13 replicates; light green lines required 10 of 13. Solid lines correspond to the number of SNPs passing the QC filter at the particular cut-off value; dotted lines correspond to the PFP. For all values of the filter cut-off, requiring fewer replicates to pass retained a greater number of SNPs. At cut-off values of 0.9 to 0.95 the PFP increased, perhaps reflecting the removal of real associations. To optimize both PFP and SNP coverage, either a cut-off value of 0.8, requiring 11 of 13 passing replicates, or a cut-off value of 0.85, requiring 12 of 13 passing replicates, may be appropriate. Here we adopted a cut-off threshold of 0.8 and required a pass rate of 80% among the replicates. (0.64 MB TIF) [file pgen.1000866.s006.tif]
