# Supplementary material for: Rapid Assessment of Genetic Ancestry in Populations of Unknown Origin by Genome-Wide Genotyping of Pooled Samples
Source: PLoS Genet. 2010 Mar 5;6(3):e1000866. doi: 10.1371/journal.pgen.1000866 (PMC2832667; doi:10.1371/journal.pgen.1000866)
Supplement: Table S1 — FST between pooled panels and their respective pseudopopulations. (0.03 MB DOC) [file pgen.1000866.s007.doc]

| Pool | F_ST_ | Standard Error |
| --- | --- | --- |
| MAY | 0.00162 | 2.11x10^-5^ |
| GXE | 0.00145 | 1.95x10^-5^ |
| SPT | 0.00221 | 3.07x10^-5^ |
| MEC-L | 0.00880 | 1.07x10^-4^ |
| MEC-H | 0.00995 | 1.21x10^-4^ |

**Table S1:** F_ST_ between pooled panels and their respective pseudopopulations.

F_ST_ was estimated according to Wright’s approximate formula, F_ST_ = (H_T_ – H_S_) / H_T_ (see Text S1) for each of 12,000 random SNPs post-QC in all pools, and then averaged to obtain the overall value shown here. Standard error was calculated as the standard deviation / √12,000. Increasing values of FST from the Jamaican samples to the Latina and Hawaiian samples corresponded with the relative degree of rightward shift in AF difference distribution seen in Figure 2.
