# Supplementary material for: Rapid Assessment of Genetic Ancestry in Populations of Unknown Origin by Genome-Wide Genotyping of Pooled Samples
Source: PLoS Genet. 2010 Mar 5;6(3):e1000866. doi: 10.1371/journal.pgen.1000866 (PMC2832667; doi:10.1371/journal.pgen.1000866)
Supplement: Text S1 — Supplemental methods. (0.07 MB DOC) [file pgen.1000866.s009.doc]

**Text S1. Supplemental Methods**

**Study populations**

The cohorts used in this study consisted of 775 African American individuals from Maywood, IL (MAY); 1,039 and 1,467 Jamaican individuals from Kingston (GXE) and Spanishtown (SPT), Jamaica, respectively; and 391 African American (MEC-AA) women, 298 Native Hawaiian (MEC-H) women, 363 Latin American (MEC-L), and 202 Japanese American women (MEC-J) from the Multi-Ethnic Cohort (MEC) of Los Angeles and Hawaii [1].

DNA samples for the MAY panel were obtained from a larger cohort of families enrolled in studies of blood pressure at Loyola University in Maywood, IL, a working-class neighborhood adjacent to Chicago, IL. The survey enrolled a representative random sample of the population between the ages of 18-74, regardless of obesity phenotype. This subcohort of 775 individuals was selected as apparently unrelated participants and oversampled at the upper and lower ends of the BMI distribution (H.N.L., R.S.C., X.Z., and J.N.H., unpublished).

DNA samples for the GXE panel were obtained from a survey conducted in the capital city of Jamaica, Kingston, as part of a larger project to examine gene by environment interactions in the determination of blood pressure among adults 25-74 years of age. The principal criterion for eligibility was a body mass index (BMI) in either the top or bottom third of BMI for the Jamaican population [2]. Participants were identified principally from the records of the Heart Foundation of Jamaica, a non-governmental organization based in Kingston, which provides low-cost screening services (height and weight, blood pressure, glucose, cholesterol) to the general public. Other participants were identified from among participants in family studies of blood pressure at the Tropical Metabolism Research Unit (TMRU) and from among staff members at the University of the West Indies, Mona. All participants were unrelated. A total of 1,039 persons were enrolled.

DNA samples for the SPT panel were obtained from a survey conducted as part of the International Collaborative Study of Hypertension in Blacks (previously described in detail in [2]). In this study a stratified random sample of the Jamaican population aged 25-74 years was recruited from in and around Spanish Town, a stable, residential urban area neighboring the capital city of Kingston. The 1,467 samples in the SPT panel were those available at the time of this study from among 2,096 participants enrolled between 1993 and 1998.

DNA samples for the MEC panels were chosen as part of a separate study examining the timing of puberty from the larger MEC cohort and represent women with either early (< 11 years of age) or late (> 14 years of age) age at menarche [1,3].

**DNA pool construction and genotyping**

As the original aim of the study was to assess the feasibility of GWA studies using pooled DNA to identify variants associated with complex human traits, pools were constructed in a case/control manner. However, for the purposes of the present study, each pool from a given panel was treated as an independent sample of the same underlying population, as no systematic difference in allele frequency is expected between individuals with different phenotype status.

In the MAY and SPT panels, individuals with BMI < 25 were designated as unaffected controls and individuals with BMI > 30 were designated as affected cases. In the GXE panel, individuals with BMI < 24 were designated as controls, and individuals with BMI > 28 were designated as cases. Males and females were pooled separately, but using the same BMI cutoffs. Before pooling, we made 1:10 dilutions of stock DNA samples to obtain diluted DNAs with approximate concentrations of 3.5 ng/ul. DNA concentrations were determined by PicoGreen (Molecular Probes, Eugene, OR) and used to determine the volume necessary to obtain 25 ng of DNA for each sample. Samples with concentrations too low to obtain 25 ng DNA in the available volume were excluded from the pools. Final sizes for the MAY pools were 114 case females, 161 control females, 101 case males, and 145 control males. Final sizes for the GXE pools were 101 case males, 79 control males, 136, 137, and 137 case females, and 98 control females. Final sizes for the SPT pools were 105 and 111 control females and 129 and 135 case females. The MEC samples were pooled by early (< 11 years of age) menarche or late (> 14 years of age) menarche. The final MEC pools consisted of 321 MEC-AA individuals (153 late menarche and 168 early menarche), 252 MEC-H individuals (120 late and 132 early), and 332 MEC-L individuals (163 late and 169 early). Once the pools were constructed, they were then concentrated to an acceptable range for Affymetrix genotyping (20-50 ng/ul) using a Speed-Vac.

Pools were genotyped using the Affymetrix Genome-Wide Human SNP 6.0 Array according to the manufacturer’s instructions (<http://www.affymetrix.com/support/technical/byproduct.affx?product=genomewidesnp_6>). The DNA pools went through the initial chemistry along with the MAY individual DNA samples and were genotyped on the same plates as the MAY samples, which were undergoing a separate GWA study for obesity. Each pool was genotyped on three arrays. Replicates with excessively low intensity (< 400u), low call rate (< 70%), or high heterozygosity compared to the other replicates within the same pool (> 5% above average of the other replicates) were deemed to be of poor quality. One of the MEC-L late replicates, one of the MEC-J early replicates, and one of the MAY case female replicates genotyped poorly, and thus each replicate was re-genotyped along with a companion replicate (one replicate from the early MEC-L pool, one from the late MEC-J pool, and one from the MAY control female pool) on a later plate. One replicate from each of three different pools (GXE control males, GXE case females, and SPT control females) also genotyped poorly and was dropped from the study.

**SNP quality control filters**

*Separation of clusters (FLD-filter).* Using parameters from each of the three genotype clusters as output by Birdseed, including the covariance matrices, we measured separation of clusters using Fisher’s linear discriminant (FLD), given by:

$FLD=\frac{\left[w^{T}\left(mean_{1}-mean_{2}\right)\right]^{2}}{w^{T}\left(\mathrm{cov}_{1}+\mathrm{cov}_{2}\right)w}$,

where$w=\left[inv\left(\mathrm{cov}_{1}+\mathrm{cov}_{2}\right)\times\left(mean_{1}+mean_{2}\right)\right]$, *w^T^* is the transpose of *w*, and *cov_i_* is the 2D-covariance matrix of cluster *i* and *mean_i_* is the vector form of the means (*i.e.*, the coordinates of the centroid) of cluster *i*. A high FLD value represents good confidence in distinguishing the individual genotype clusters, and as such should yield a more accurate estimate of the pooled allele frequency. Given three genotype clusters, there are two FLD values for each SNP on each plate, and the minimum of the two values was used in filtering to give a conservative estimate of SNP performance. PFPs were calculated using FLD cut-offs of 16, 20, and 25, with FLD = 20 selected as the final cut-off (Figure S4A).

*Radius of pool intensity (r-filter).* The r-filter was calculated as a ratio of the radius (r) of the intensity of the pooled sample (*P*) to the radius (r’) of the expected intensity given the average intensities of the individual samples that make up the two closest clusters (*I*) (Figure S5). The origin (*O*) is defined as described in the Methods, and the length of the vector *OP* is the radius of the pool intensity, r. The vector *OI*, where *OI* = n*OP* for constant n, terminates at the intersection of vector *OP* and the line between centroids of the AA and AB (or the BB and AB) clusters. The length of *OI* is the radius of the expected intensity of the individual sample, r’. The r-filter is based on a ratio of the two values (r/r’). A low r/r’ ratio may reflect poor DNA quality or low DNA quantity for the pooled DNA when compared to that of the individual DNA on the same genotyping plate. Moreover, a low r/r’ ratio also subjects the estimate of pooled allele frequency to small number statistics. There is one ratio for each pool replicate at each SNP. For the current study, we considered a SNP to have passed the r-filter if at least 80% of the replicates met the minimum r/r’ = 0.8 cutoff (Figure S6).

*Population minor allele frequency (MAF-filter).* Pooled AF was estimated for each pool as described in the Methods. Pooled AF estimates from multiple pools within a population (or between cases and controls) were averaged, weighting by the number of individuals used to construct the pool. Because PFP decreased with increasing MAF-filter cut-offs, it appears that pooled AF estimates are more accurate for common SNPs (Figure S4B). Here we adopted a 5% MAF cut-off, but 10% or 15% could be appropriately adopted at the expensive of decreased SNP coverage.

*Variance of error in pooled allele frequency (hist-filter).* By taking advantage of having genotyped 521 MAY samples both individually and in pools, we were able to determine, for each SNP, the difference in the AF estimated by pooling and the actual expected AF based on individual genotyping. Across the four pools constructed using the MAY individuals, we calculated the standard deviation of this AF difference for each SNP. High variance among the differences reflects an inconsistently genotyped SNP, and may reflect SNP probes on the Affymetrix 6.0 array that are not robust to genotyping pooled DNA. Using the genomic control (GC) inflation factor [4] as a surrogate measure of proportion of false positive associations, inconsistently genotyped SNPs as defined here appear to represent SNPs that perform poorly in general (Figure S4C). We adopted a standard deviation of 0.06 as the historical (as this filter is based on historical data) filter cut-off. This filter is platform-specific and not study-specific, and thus as additional cohorts are genotyped as pools and individuals on the Affymetrix 6.0 array, the historical filter can be adjusted accordingly to reflect newly available data.

**Estimation of admixture proportion using STRUCTURE**

To estimate admixture proportion using genotypes at known ancestry informative markers in the MAY pool, we performed “supervised” STRUCTURE analysis, using STRUCTURE version 2.2.3 for UNIX [5]. The default main parameters and extra parameters were used, with the following exceptions: for the main parameters, POPFLAG was set to 1, PHENOTYPE to 0, MISSING to 0, ONEROWPERIND to 1, and BURNIN and NUMREPS to 10,000; for extra parameters, FREQSCORR was set to 0, POPALPHAS to 1, USEPOPINFO to 1, GENSBACK to 2, PFROMPOPFLAGONLY to 1, and ALPHAMAX to 20.0. *k* (the number of expected ancestral populations) was set at 2. Genotypes for the HapMap YRI and CEU populations were used for prior population information to assist clustering. Genotypes at 699 AIM loci for 740 MAY individuals were used to compute admixture proportion in the MAY population.

Admixture proportions for MEC-H and MEC-L pools had been previously determined using STRUCTURE and 69 AIMs [3].

**Principal component analysis (PCA) using EIGENSOFT**

HapMap phase 3 data were downloaded from www.hapmap.org in PLINK format, and converted to the EIGENSTRAT format using the default parameters of the convertf utility in the EIGENSOFT package [6]. PCA was carried out using the smartpca utility with the default parameters, with LD correction turned on (nsnpldregress = 2, as recommended by the EIGENSOFT authors).

**Calculation of F_ST_**

F_ST_ was estimated according to Wright’s approximate formula, F_ST_ = (H_T_ – H_S_) / H_T_, where H_T_ is the expected heterozygosity per locus of the overall total population and H_S_ is the expected heterozygosity per locus in the subpopulations [7]. Populations were assumed to be in Hardy-Weinberg Equilibrium and the number of heterozygotes was calculated as 2p(1-p), where p is the estimated AF from the pooled panel or the weighted AF from the corresponding pseudopopulation. The pseudopopulation for each pooled panel was constructed as described in the Methods, using the admixture proportions estimated in Table 1 (pooling method). An F_ST_ value was calculated for each of 12,000 random SNPs passing QC filtering (see Methods and Text S1) in all pools listed, and then averaged. Standard error was calculated as standard deviation divided by 12,000.

**Technical validation by individual genotyping**

25, 28, and 26 candidate AIMs in MEC-L, GXE and SPT, respectively were successfully genotyped in the individuals comprising the pools. Genotyping was performed as described elsewhere [8], using the Sequenom MassARRAY platform [9] with the iPLEX protocol. The basic protocol involves a multiplex primer extension followed by matrix-assisted laser desorption ionization-time of flight mass spectroscopy detection [9]. SNPs were considered working if they genotyped successfully in 90% or more of the samples and had no more than one consensus error among 32 triplicates in SPT and 34 triplicates in GXE (for a total of 96 and 102 internal comparisons per SNP, respectively) or among 36 internal comparisons for MEC-L. SNPs were evaluated by these parameters in each cohort independently.

**References**

1. Kolonel LN, Henderson BE, Hankin JH, Nomura AM, Wilkens LR, et al. (2000) A multiethnic cohort in Hawaii and Los Angeles: baseline characteristics. Am J Epidemiol 151: 346-357.

2. Cooper R, Rotimi C, Ataman S, McGee D, Osotimehin B, et al. (1997) The prevalence of hypertension in seven populations of west African origin. Am J Public Health 87: 160-168.

3. Gajdos ZK, Butler JL, Henderson KD, He C, Supelak PJ, et al. (2008) Association studies of common variants in 10 hypogonadotropic hypogonadism genes with age at menarche. J Clin Endocrinol Metab 93: 4290-4298.

4. Devlin B, Roeder K (1999) Genomic control for association studies. Biometrics 55: 997-1004.

5. Pritchard JK, Stephens M, Donnelly P (2000) Inference of population structure using multilocus genotype data. Genetics 155: 945-959.

6. Price AL, Patterson NJ, Plenge RM, Weinblatt ME, Shadick NA, et al. (2006) Principal components analysis corrects for stratification in genome-wide association studies. Nat Genet 38: 904-909.

7. Wright S (1950) Genetical structure of populations. Nature 166: 247-249.

8. Gabriel SB, Schaffner SF, Nguyen H, Moore JM, Roy J, et al. (2002) The structure of haplotype blocks in the human genome. Science 296: 2225-2229.

9. Tang K, Fu DJ, Julien D, Braun A, Cantor CR, et al. (1999) Chip-based genotyping by mass spectrometry. Proc Natl Acad Sci U S A 96: 10016-10020.
