# Supplementary material for: Rapid Assessment of Genetic Ancestry in Populations of Unknown Origin by Genome-Wide Genotyping of Pooled Samples
Source: PLoS Genet. 2010 Mar 5;6(3):e1000866. doi: 10.1371/journal.pgen.1000866 (PMC2832667; doi:10.1371/journal.pgen.1000866)
Supplement: Text S2 — Formula derivations. (0.06 MB DOC) [file pgen.1000866.s010.doc]

**Text S2**

**Deriving the angle *θ*measuring the degree of rotation with respect to the horizontal axis of a cluster of data points with error.**

Start with a function describing the ellipse:

$\frac{x^{2}}{a^{2}}+\frac{y^{2}}{b^{2}}=1$, for constants a and b.

If the ellipse is rotated, then x 🡪 xcos(*θ*) + ysin(*θ*) and y 🡪 ycos(*θ*) - xsin(*θ*).

By substitution, the equation for the ellipse becomes:

$x^{2}\left(\frac{\cos^{2}\text{(}\theta\text{)}}{a^{2}}+\frac{\sin^{2}\text{(}\theta\text{)}}{\text{b}^{2}}\right)+2xy\cos(\theta)\sin(\theta)\left(\frac{1}{a^{2}}-\frac{1}{b^{2}}\right)+y^{2}\left(\frac{\sin^{2}(\theta)}{a^{2}}+\frac{\cos^{2}(\theta)}{b^{2}}\right)=1$ (1).

The distance of a point$\vec{x}$= (x_1_, x_2_, x_3_, …)^T^ to a cluster of points with mean$\vec{\mu}$= (μ_1_, μ_2_, μ_3_,..)^T^ in units of$\sigma^{2}$is given by the Mahalanobis distance:

(2),

where C^-1^ is the inverse of the covariance matrix.

Taking a 2-dimensional scenario where$\vec{x}$ = (x, y)^T^, $\vec{\mu}$ = (0,0)^T^, and

$$C^{-1}=\frac{1}{\det C}\left[\begin{matrix} C_{yy} & -C_{xy} \\ -C_{xy} & C_{xx} \end{matrix}\right]$$

without loss of generality, multiplying out equation (2) results in the equation for an ellipse:

$D^{2}\left(\left(\begin{matrix} x \\ y \end{matrix}\right)\right)=x^{2}\frac{C_{yy}}{\det C}+2xy\frac{-C_{xy}}{\det C}+y^{2}\frac{C_{xx}}{\det C}$ (3).

By taking a point 1$\sigma^{2}$away from the error ellipse (D^2^(x,y) = 1), we identify from equation (1):

$\frac{C_{yy}}{\det C}=\frac{\cos^{2}\theta}{a^{2}}+\frac{\sin^{2}\theta}{b^{2}}$ (4a),

$\frac{C_{xx}}{\det C}=\frac{\sin^{2}\theta}{a^{2}}+\frac{\cos^{2}\theta}{b^{2}}$ (4b), and

$\frac{-C_{xy}}{\det C}=\sin(\theta)\cos(\theta)\left(\frac{1}{a^{2}}-\frac{1}{b^{2}}\right)=\frac{1}{2}\sin(2\theta)\left(\frac{1}{a^{2}}-\frac{1}{b^{2}}\right)$ (4c).

By applying the double-angle identity (cos2*θ* = cos^2^*θ* – sin^2^*θ*) and subtracting equation (4b) from equation (4a), after some algebra, we get:

$\frac{1}{\det C}\left(\frac{C_{yy}-C_{xx}}{\cos2\theta}\right)=\left(\frac{1}{a^{2}}-\frac{1}{b^{2}}\right)$ (5).

Finally, by substituting equation (5) into equation (4c) and performing some algebraic manipulations, we recover the identity shown in the Methods:

$$\theta=\frac{1}{2}\arctan\left(\frac{2C_{xy}}{C_{xx}-C_{yy}}\right)$$

**The intercept in the regression model for estimating proportion of admixture is half of the unexplained ancestry.**

In a two population regression model, the allele frequency of the unknown population is modeled as:

$P_{ui}=c+\beta_{1}P_{1i}+\beta_{2}P_{2i}$,

where *P_ui_* is the estimated allele frequency from pooling in the population of unknown admixture for SNP *i*, *P_1i_* and *P_2i_* are the allele frequencies in the ancestral (reference) populations 1 and 2 for SNP *i*, and c is the regression intercept.

However, a better model including a third ancestral population would be:

$P_{ui}=\beta_{1}P_{1i}+\beta_{2}P_{2i}+(1-\beta_{1}-\beta_{2})P_{3i}$.

Thus $c=(1-\beta_{1}-\beta_{2})P_{3i}$. By taking the expectation on both sides:

$E\left(c\right)=(1-\beta_{1}-\beta_{2})E\left(P_{3i}\right)$ over all SNP *i*.

$E\left(P_{3i}\right)=0.50$ over all SNP *i*, as the allele frequency for SNP *i* is with respect to allele A according to the Affymetrix 6.0 array annotation (<http://www.affymetrix.com/support/technical/annotationfilesmain.affx>, GenomeWideSNP_6_Annotations, na25), and allele A annotation is independent of the minor allele of each SNP. Therefore:

$E\left(c\right)=\frac{1}{2}(1-\beta_{1}-\beta_{2})$,

*i.e.,* the regression intercept is half of the unexplained ancestry in the model.
